# P03 Amikacin drug levels and efficacy—a 3 month remissive study

**DOI:** 10.1093/jacamr/dlad143.007

**Published:** 2024-01-03

**Authors:** Ana Coutinho

**Affiliations:** Hospital de Cascais – Dr José de Almeida, Alcabideche, Portugal

## Abstract

**Background:**

The therapeutic drug monitoring (TDM) of toxic drugs is a routine practice in our 200-bed hospital. Amikacin is not often used except in MDR bacteria. TDM is based on trough and peak levels of aminoglycosides, although AUC TDM may be the way forward, as discussed in the conclusions. According to the literature, measurement of serum amikacin levels is indicated in cases when prolonged aminoglycoside therapy is expected or in patients with compromised renal function and is not recommended for aminoglycoside use for less than 5–7 days or for treatment of mild infections in patients with no risk factors for drug toxicity.

**Objectives:**

This study delivers some controversy on not to recommend monitoring for aminoglycoside use for less than 5–7 days, or for treatment of mild infections in patients with no risk factors for drug toxicity.

**Methods:**

A retrospective study was conducted in our hospital between May and June 2023. Five patients aged 62–82 years on amikacin therapy for 5 to 10 days were included, all with *Klebsiella pneumoniae* OXA-48 UTI. At least two peak and trough samples were collected, and data were submitted to the Precise PK^©^ software for precision dosing. The software is designed to predict population-based levels and patient-based levels. Interpretation of these values by pharmacists delivers optimized/viable amikacin intervals and dose individualized recommendations.

**Results:**

Results are shown in Table 1.

**Conclusions:**

Serum amikacin monitoring should guide therapy to ensure adequate levels, especially in Gram-negative resistant bacterial infections. According to these results, with Gram-negative resistant bacteria, it is worth monitoring even the short treatments—as seen in Patient 3. AUC TDM instead of peak TDM might be the way forward— this obviates distribution phase underdosing and delivers more accurate correlation to efficacy after changing intervals.

**Table 1.** Results

Serum Cr and co-factors	Initial amikacin prescription	First drug level, Day 3	Dose/dosing interval adjustment	Second drug level, Day 4 or 5	DOT^+^/outcome
Patient 1^+^* Cr 0.98	500 mg 12/12 h	Toxic trough: 7	500/24 h	Trough: 0.88, peak: 40	10 days/positive outcome
Patient 2^+^* Cr 2.4	500 mg 72/72 h**	Therapeutic	No adjustment	Therapeutic	10 days/positive outcome
Patient 3^+^ Cr 0.7	1000 mg 48/48 h***	Sub therapeutic peak: 20	1250 48/48 h	Trough: 0.33, peak: 39	7 days/positive outcome
Patient 4^+^ Cr 0.7	500 mg 12/12 h	Therapeutic	No adjustment	Therapeutic	10 days/negative outcome
Patient 5 Cr 1.36	1500 mg/24 h	Therapeutic	No adjustment	Therapeutic	5 days/positive outcome

+Associated with meropenem.

*Urinary catheter.

**Previous ceftazidime/avibactam failure.

***Ambulatory patient.

